# Usutu Virus Antibody Dynamics in Naturally Infected Blackbirds, the Netherlands, 2016–2018

**DOI:** 10.3201/eid3106.241744

**Published:** 2025-06

**Authors:** Erwin de Bruin, Jurrian van Irsel, Felicity Chandler, Robert Kohl, Tess van de Voorde, Anne van der Linden, Fred de Boer, Marion Koopmans, Henk van der Jeugd, Chantal Reusken

**Affiliations:** ErasmusMC, Rotterdam, the Netherlands (E. de Bruin, F. Chandler, R. Kohl, A. van der Linden, M. Koopmans, C. Reusken); Nederlands Instituut voor Ecologie, Dutch Centre for Avian Migration and Demography, Wageningen, the Netherlands (J. van Irsel, T. van de Voorde, H. van der Jeugd); Wildlife Ecology and Conservation Group, Wageningen University and Research, Wageningen (W.F. de Boer)

**Keywords:** Usutu virus, West Nile virus, viruses, zoonoses, arboviruses, antibodies, vector-borne infections, blackbirds, Turdus merula, the Netherlands

## Abstract

Usutu virus is a zoonotic arbovirus that causes massive mortality in blackbirds. Using a unique longitudinal dataset on the kinetics of virus-specific antibodies in naturally infected wild blackbirds (*Turdus merula*), we found that individual birds may remain seropositive for >1 year and that reinfection can occur despite low-level virus neutralizing antibodies.

Usutu virus (USUV), a mosquitoborne zoonotic virus of the genus *Orthoflavivirus*, was first detected in the Netherlands in 2016 (*1*). Eurasian blackbirds (*Turdus merula*) are remarkably susceptible to USUV disease; exceptional die-off among blackbirds is a hallmark of the (re)-emergence of virus circulation in a region (*2*,*3*). Information on survival of blackbirds after USUV infection and the resulting prevalence of USUV antibodies in such populations is limited, and knowledge of the longitudinal dynamics in USUV antibody responses in individual naturally infected blackbirds is lacking. We studied the presence of USUV-specific antibodies in blackbirds during the first 3 years after the initial detection of the virus in the Netherlands. 

During March 2016–October 2018, as part of a broader study of zoonoses in songbirds in the Netherlands, we registered 1,181 blackbird captures; 969 unique birds were captured 1–9 times (*1*,*4*). Sampling locations were biased; of 77 sampling locations, just 4 contributed nearly 50% of all samples (Overdinkel, 52°14′N, 7°01′E; Wageningen, 51°59′N, 5°39′E; Haarzuilens, 52°08′N, 5°00′E; Eastermar, 53°11′N, 6°03′E). 

We sampled healthy birds by wing vein puncture. Overall, a total of 667 serum samples from 534 unique blackbirds were tested. The samples were screened in a 2-tier system of protein microarray testing including the nonstructural 1 proteins of USUV and West Nile virus (WNV), followed by confirmation of positive IgG results based on comparative USUV and WNV neutralization (*5*). USUV and WNV belong to the same serogroup, causing probable cross-reactivity in serologic assays (*6*). Therefore, we only considered serum samples confirmed for antibodies when microarray reactivity was confirmed by comparative virus neutralization test (VNT) results showing USUV antibody titers >4-fold higher than a WNV antibody titer, or vice versa. Serum samples with a positive USUV microarray result but no conclusive confirmation by VNT were considered orthoflavivirus positive (Table). To determine the USUV seroprevalence by calendar year, we only included birds that were repeatedly sampled within the same year once, and in cases of seroconversion within a year (n = 4), we only included the positive outcome. 

**Table:** Summary of serologic results for USUV and WNV in a study of antibody dynamics in naturally infected blackbirds, the Netherlands, 2016–2018*

Serologic result	USUV		WNV VNT	No. samples			
	Protein microarray	VNT		2016	2017	2018	Total
USUV negative	<6,000	NT	NT	59	249	192	500
USUV positive	>6,000	>16†	4-fold <USUV VNT	3	21	26	50
WNVpositive	<6,000	<16	>16	0	0	1	1
Subtotal				62	270	219	551
Orthoflavivirus positive	>6,000	>16	NT	0	7	9	16
	>6,000	>16 equal to WNV VNT	>16 equal to USUV VNT	0	0	3	3
	<6,000	NT	>16	0	0	2	2
Subtotal				0	7	14	21
Possible orthoflavivirus	>6,000	NT	NT	1	3	4	8
Subtotal				1	3	4	8
Total				63	280	237	580

*Serum samples were screened in a 2-tier system of protein microarray testing, followed by confirmation of positive IgG results based on VNT. Strains used for virus neutralization were WNV strain B956 and USUV isolate Africa-3 collected from a blackbird in the Netherlands in 2016. Serum was only considered confirmed for USUV antibodies when VNT USUV antibody titer was >4-fold the WNV antibody titer, and vice versa for WNV. Serum samples with a positive USUV microarray result but no conclusive confirmation by VNT were considered orthoflavivirus positive. Negative indicates absence of detectable level of specific antibodies, positive indicated presence of detectable level of specific antibodies. NT, not tested; USUV, Usutu virus; VNT, virus neutralization test; WNV, West Nile virus. †Reciprocal titers ranged from 1:16 to >1:1,024.

According to the stated criteria for confirmed infection, 3 (4.8%) of 63 unique birds were positive for USUV-specific antibodies in 2016; in 2017, the total was 21 (7.5%) of 280; and in 2018, the total was 26 (10.0%) of 237 (Table). Results showed a significant increase in seroprevalence over those years [χ^2^(1) = 6.01, p = 0.01; Hosmer-Lemeshow goodness-of-fit χ^2^(8) = 3.24, p = 0.92], with a peak in autumn 2017 (data not shown) (*7*). That increase is in line with observations in Austria, where seropositivity of 9.1% in blackbirds was found 2 years after the virus was first detected, and 9.6% 3 years after (*8*). However, in Germany, seropositivity remained low (<3.9%) in the 5 years after a massive blackbird die-off (*9*). 

Starting in September 2016, the Netherlands study captured 124 blackbirds >1 time (range 2–9 times); 84 birds had serum samples taken at >1 capture. Of those birds, 17 were confirmed seropositive >1 time; 7 seroconverted during the study period, and 10 were positive at all time points (Figure, panel A). Of the remaining 67 recaptured birds, 66 remained seronegative throughout the study, whereas 1 bird seroconverted for USUV, based on microarray and USUV VNT only. No seroreversion was observed. 

**Figure:**
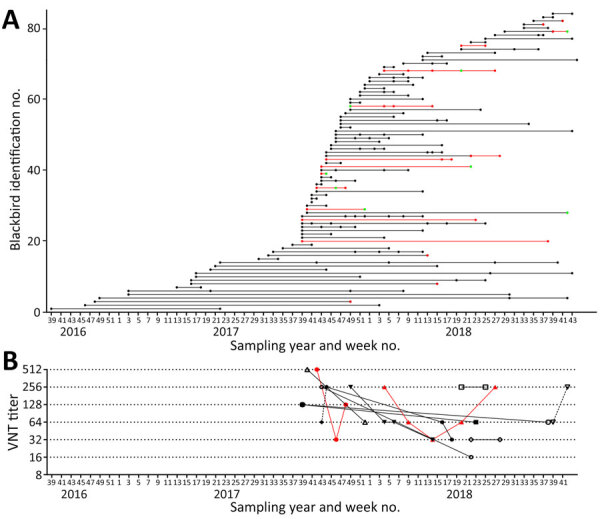
Longitudinal results for Usutu virus (USUV) serologic testing of serum samples from naturally study of USUV antibody dynamics in naturally infected blackbirds, the Netherlands, 2016–2018. Birds were recaptured and sampled >1 time. A) USUV seropositivity status for recaptured blackbirds that were sampled 2–9 times (n = 84). Left axis indicates individual blackbird identification numbers (1–84); dots represent moment of actual sampling; lines connecting sampling moments of each individual bird are supportive to figure interpretation and have no meaning. Black dots indicate seronegative by microarray; red dots, confirmed positive for USUV antibodies by microarray and comparative VNT USUV versus West Nile virus; green dots, confirmed positive for orthoflavivirus antibodies. B) Trends in USUV virus neutralization test titers in time for recaptured blackbirds that were seropositive >1 time (n = 11). Reciprocal antibody titers are shown. Red lines indicate birds that showed an increase in titer after an initial decline; black lines indicate birds that showed no kinetics or a decline in titer over time. VNT, virus neutralization test.

The longest period of seropositivity recorded was >361 days. That bird was already seropositive at first sampling and was not followed beyond 361 days. To gain insight into functional neutralizing antibody kinetics, we plotted over time the VNT titers of the 12 birds that were positive at multiple sampling points (Figure, panel B). Six birds showed waning neutralizing antibody titers in time with variable kinetics. Two birds showed no changes in titers in samples taken 33 and 42 days apart, whereas 2 other birds showed an increase in titers taken 5 and 21 days apart. Two birds showed an initial decline in VNT titers, then an increase within 41 and 113 days; corresponding cloaca swab specimens showed a similar trend (positive-negative-positive) for the presence of USUV RNA (no sequence information available). That finding might be indicative for the occurrence of reinfection in the presence of low USUV-neutralizing antibodies, whether or not by different USUV lineages; Africa-3 is the most common infecting lineage in the Netherlands, followed by Europe-3 lineage (*4*). Alternatively, such observations might be the result of exacerbation of a persistent infection (*10*). Conclusive evidence is needed from additional longitudinal studies, including experimental infections. Further insight into the longevity of USUV-specific antibodies in different bird species, the occurrence of reinfections, and the relationship between variations in the immune-status of a specific bird population and variations in the level of virus circulation will contribute to a better understanding of the added value of the assessment of public health risks based on seroecologic surveillance. 

In conclusion, our study provides a unique longitudinal dataset on the kinetics of USUV-specific antibodies in naturally infected wild blackbirds. We show seropositivity in individual blackbirds for >1 year and the possible occurrence of reinfection in the presence of low-level virus neutralizing antibodies.
